# Age-related deficits in bilateral motor synergies and force coordination

**DOI:** 10.1186/s12877-019-1285-x

**Published:** 2019-10-24

**Authors:** Nyeonju Kang, Lisa M. Roberts, Clara Aziz, James H. Cauraugh

**Affiliations:** 1 Division of Sport Science and Sport Science Institute, Incheon, South Korea; 2 Neuromechanical Rehabilitation Research Laboratory, Incheon National University, Incheon, South Korea; 3 Department of Applied Physiology and Kinesiology, University of Florida, Gainesville, FL 32611-8206 USA; 4 Department of Medicine, University of Alabama at Birmingham, Birmingham, AL USA

**Keywords:** Ageing, Uncontrolled manifold, Interlimb coordination, Motor control, Visual information

## Abstract

**Background:**

Ageing may cause impairments in executing bilateral movement control. This study investigated age-related changes in interlimb force coordination across multiple trials by quantifying bilateral motor synergies based on the uncontrolled manifold hypothesis. Participants completed the trials with and without visual feedback.

**Methods:**

Twenty healthy individuals (10 older adults and 10 young adults) performed 12 isometric force control trials for the two vision conditions at 5% of maximal voluntary contraction. All dependent variables were analyzed in two-way mixed model (Group × Vision Condition; 2 × 2) ANOVAs with repeated measures on the last factor.

**Results:**

The analyses revealed that older adults had greater mean force produced by two hands in both vision conditions (i.e., yes and no visual feedback). Across both vision conditions, the older adult group showed greater asymmetrical force variability (i.e., standard deviation of non-dominant hand > standard deviation of dominant hand) and revealed more positive correlation coefficients between forces produced by two hands as compared with the young adult group. Finally, an index of bilateral motor synergies was significantly greater in young adults than older adults when visual feedback was available.

**Conclusion:**

The current findings indicate that deficits in interlimb force coordination across multiple trials appeared in older adults.

## Background

Ageing typically causes deficits in executing and controlling various movements of the upper extremities because of physiological alterations in the neuromuscular system [1, 2]. Importantly, upper limb dysfunction in older adults interferes with activities of daily living and may be an index for motor and cognitive impairments [3–5]. Thus, estimating capabilities to modulate upper limb movements may effectively indicate one’s ageing progression.

Interlimb coordination, executing cooperative actions between two hands, is one of the critical functions of the upper extremities for older adults contributing to successfully performing their fundamental movements of daily living [6, 7]. However, older adults frequently revealed significant impairment in motor coordination between hands [6], and these deficits were correlated with the appearance of age-related cognitive diseases [5, 8]. A traditional way to quantify interlimb coordination functions is to test bilateral isometric force control [9, 10]. During this bilateral force control task, a performer attempts to match and continue the sum of forces produced by the two hands around a submaximal targeted force level. Then, calculating correlation coefficients on two individual force signals from each hand indicates the strength of interlimb coordination [9, 11]. A higher frequency of positive correlation coefficients (close to 1) indicates a symmetric and an in-phase pattern that produces similar forces between hands. Alternatively, a higher frequency of negative correlation coefficients (close to − 1) denotes an asymmetric and an anti-phase pattern that produces differential forces between hands. Newell and colleagues reported that healthy young adults tended to show more negative correlations between left and right hand forces, and these coordination patterns contributed to improved bilateral force control performance [9, 12]. The authors suggested that the anti-phase behaviors between hands might indicate an individuals’ error-compensatory strategy.

The correlation coefficient between forces produced by two hands indicated abnormal interlimb coordination function in older adults [12–14]. As compared with young adults, during bilateral force control older adults showed more positive correlation coefficients indicating that their two hands produced forces in the same direction [9]. Moreover, during a bilateral grip force coordination task requiring timing control while transferring force production between two hands, the older adult group produced longer alternating intervals in the dominant to non-dominant hands force transition condition as well as in the non-dominant to dominant hands force transition condition [13]. However, these findings were limited to estimating cooperative behaviors reflecting only online motor corrections within a single-trial [14]. Given that how individuals select and plan appropriate motor actions across trials is a crucial process that may require a higher level of cognitive control than motor correction within a single-trial [15, 16], addressing whether ageing interferes with an individual’s interlimb coordination adjustments across multiple trials is an interesting approach for additional ageing information.

According to the uncontrolled manifold (UCM) hypothesis advocated by Latash and colleagues, the human central nervous system (CNS) selects an appropriate motor solution from numerous alternatives. Thus, the CNS tends to organize multiple combinations of the effectors in a synergic way each of which is equally capable of achieving the appropriate task goal rather than searching for a unique solution [17–19]. The number of motor solutions contributing to solving a task are uncontrolled, and these combinations denote motor synergies. Recent studies used the UCM hypothesis for estimating interlimb coordination patterns across multiple trials during bilateral force control [10, 18, 20, 21]. The UCM analysis posited two sub-spaces: (a) the UCM line corresponding to perfect task performance and (b) the ORT line orthogonal to the UCM line corresponding to task errors. Further, the analysis considered mean forces produced by two hands for each trial as a pair referred to as the fundamental element. The variance of the fundamental element pairs across multiple trials projected to the UCM line denoted good variability because this variability positively contributed to task performance. In contrast, the variance of the fundamental element pairs projected to the ORT line indicated bad variability because this variability interfered with task performance. Thus, an index of the bilateral motor synergies was a proportion of good variability relative to bad variability. Bilateral motor synergies reflecting a higher proportion of good variability than bad variability indicated superior coordination functions across trials. Although a recent UCM study reported less bilateral motor synergies in older adults during a two-foot ankle force control task [21], these altered interlimb coordination patterns across multiple trials were restricted to impaired bilateral lower limb control. Given the importance of upper limb control in successful activities of daily movements of older adults, determining how ageing influences bilateral upper limb control in a synergic way is still necessary.

In addition, the manipulation of visual feedback may influence interlimb coordination function in older adults because ageing may interfere with visuomotor processing. Previous studies reported that a higher visual gain (e.g., more visual feedback) impaired both unilateral force control [22] and bilateral coordination capabilities in older adults [12]. Furthermore, across vision and no vision conditions, older adults showed greater force variability during unilateral force control tasks while simultaneously processing visual information than young adults, whereas no differences were found between the two groups in the no vision condition [23, 24]. Thus, the purpose of this study was to investigate age-related changes in interlimb coordination as indicated by bilateral motor synergies during isometric force control for two visual conditions: yes and no visual feedback. Based on prior findings [23, 24], we hypothesized that older adults would show less bilateral motor synergies than those in young adults when they performed isometric force control tasks in the visual feedback condition.

## Methods

### Ethics approval and consent

This study protocol was approved by the Institutional Review Board of the University of Florida (2017-U-01003). Each participant read and signed an approved institutional review board informed consent form prior to testing.

### Participants

Ten young (age *M* ± *SD* = 20.1 ± 1.3 years; five females and five males) and ten older adult (age *M* ± *SD* = 72.3 ± 5.0 years; two females and eight males) volunteers participated in this study. All participants were right hand-dominant healthy individuals self-reported, and we confirmed no neuromuscular disorders, orthopedic abnormalities of the fingers or hand, cognitive, or vision impairments. We conducted prior power analyses on pilot data using G*Power software (version 3.1.3). The analyses in a within-between interaction design revealed desired actual power values (≥ 0.9) at alpha = 0.05 with 20 subjects.

### Experimental setup

Consistent with prior experimental designs [10, 25], participants completed isometric force control tasks while bilaterally extending their wrist and fingers. During task execution, participants sat 78 cm away from a 43.2 cm LED monitor (1024 × 768 pixels; refresh rate = 100 Hz) and placed their left and right forearms on the desk in comfortable positions (i.e., 15-20° of shoulder flexion and 20-40° of elbow flexion). Participants placed their hands and fingers fully extended under customized platforms attached to force transducers (MLP-75, Transducer Techniques, 4.16 × 1.27 × 1.90 cm, range = 75 lbs., 0.1% sensitivity) and as we adjusted height of each platform so that the platforms barely touched the back of the knuckles of each hand. We instructed volunteers to avoid any inadvertent force production caused by elbow, shoulder, or trunk movements.

**Fig. 1:**
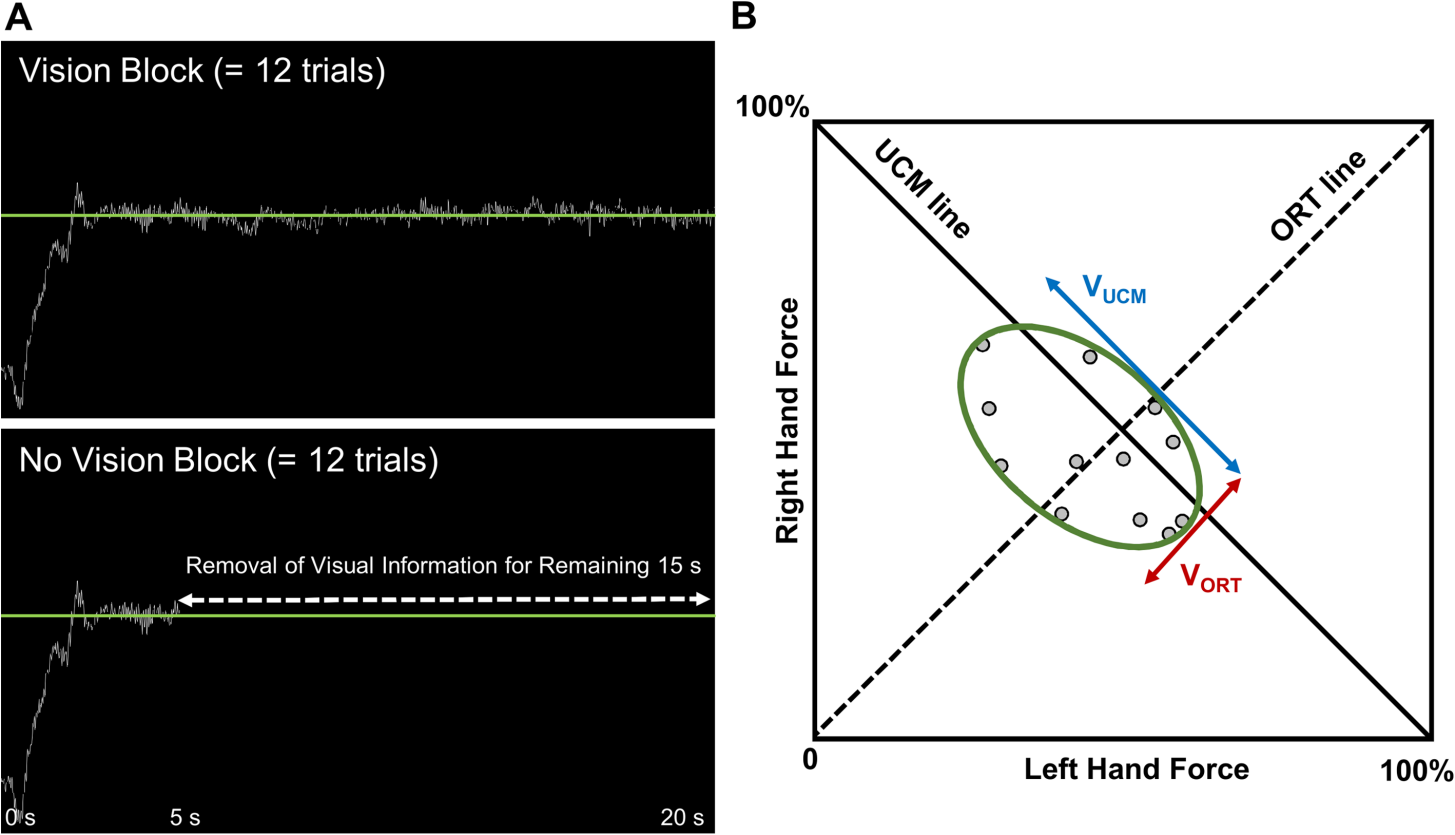
Experimental setup. **a** Visual display examples across vision conditions. **b** UCM hypothesis for calculating good and bad variability

Before testing began, we measured MVC (maximum voluntary contraction; 6 s of each trial) three times. Based on the average value of the three MVC trials, we calculated submaximal targeted force levels of each individual for the bilateral force control task. We selected 5% of MVC as a targeted force level that greatly influenced unilateral as well as bilateral force control capabilities in older adults as shown in prior findings [23, 24, 26]. During the bilateral force control task, volunteers tried to match the target force displayed and to maintain their total force (i.e., the sum of forces produced by two hands) for 20 s of each trial. Moreover, we administered two different visual feedback conditions: (a) vision and (b) no vision [23, 24, 26]. In the vision condition, the LED monitor displayed the total force with a white line and the targeted force level with a green line for 20 s. In the no vision condition, we eliminated the white line after first 5 s, and participants only saw the target force green line for the remaining 15 s (Fig. 1a). While participants completed 12 trials for each visual feedback condition, we maintained a constant 1 degree visual angle across all trials [25]. We randomly assigned the order of two conditions using a custom LabVIEW Program.

Force signals collected from force transducers were sampled at the rate of 100 Hz via a 16-bit analog-to-digital converter (A/D; NI cDAQ-9172 + NI 9215 and minimal force unit detection = 0.0016 N), and further amplified by a 15LT Grass Technologies Physio-data Amplifier System (Astro-Med Inc.) with an excitation voltage of 10 V and a gain of 200. A custom LabVIEW Program (National Instruments, Austin, USA) administered all experiments. After collecting the force data, we detrended the force signals and applied a bidirectional fourth-order Butterworth filter with 20 Hz of a cutoff frequency. Using a custom Matlab program (Math Works™ Inc., Natick, USA), we conducted offline-data analyses on all filtered force data.

### Data analyses

For 20 s of each trial, we focused on the middle 3 s (i.e., 5.0-8.0 s; 300 data points) of force signals because this phase may indicate early motor corrections immediately executed by individuals after the removal of visual information. Thus, we analyzed the force data within this middle phase across 12 trials in the vision condition and 12 trials in the no vision condition.

We calculated the mean and variability (i.e., standard deviation: SD) of total force produced by both hands for each trial. In addition, we estimated asymmetry of both mean and variability of forces between the dominant and non-dominant hands. Given that all volunteers were right-handed, we calculated the proportion of mean force produced by the left hand (i.e., non-dominant side) relative to mean force produced by the right hand (i.e., dominant side). Thus, the values of force asymmetry close to 100% indicate similar force outputs between two hands, and the values of force asymmetry greater than 100% denote more force outputs produced by the non-dominant hand than dominant hand. We used the same calculations for quantifying asymmetry of force variability.

For estimating interlimb force coordination functions, we applied two different approaches: (a) correlation coefficients and (b) bilateral motor synergies. We performed Pearson’s linear correlation analysis for calculating the correlation coefficient on force signals produced by each hand within a single-trial [9, 25]. Positive correlation coefficients (0 < *r* ≤ 1) indicate less coordinated behaviors between hands, whereas negative correlation coefficients (− 1 ≤ *r* < 0) denote better interlimb coordination function. The correlation coefficient values were Z-transformed using Eq. 1 for additional parametric statistical analyses [10, 18].

1 $$ Correlation\ Coefficient\left(Z- transformed\right)=0.5\times \mathit{\ln}\frac{2+\mathrm{r}}{2-\mathrm{r}} $$

Consistent with prior studies that examined interlimb coordination based on the UCM theory [10, 17, 18], we followed the procedure for quantifying bilateral motor synergies. First, we calculated fundamental elements for each trial (i.e., mean forces produced by the left and right hands) and normalized the raw elements using individual’s targeted force level. For example, when the performer bilaterally generated 30 N from left hand and 50 N from right hand toward 100 N of a targeted total force level for a specific trial, a pair of the normalized elements were (left hand: 30 N / 100 N × 100 = 30%, right hand: 50 N / 100 N × 100 = 50%). Then, we repeated this calculation across all 12 trials for each vision condition. Second, we projected all 12 pairs of normalized elements to two different lines, respectively: (a) the UCM line and (b) the ORT line (Fig. 1b). UCM theorists referred to the variance of elements projected to the UCM line as good variability (V_UCM_) contributing to successful motor control, whereas the variance of elements projected to the ORT line, orthogonal to the UCM line was considered as bad variability (V_ORT_) leading to impairments in motor control [17]. As shown in Eq. 2, a proportion of V_UCM_ relative to V_ORT_ serves as an index of bilateral motor synergies (V_Index_), and greater amount of V_Index_ indicates better interlimb coordination across multiple trials. Finally, all V_Index_ values were Z-transformed using Eq. 3 for additional parametric statistical analyses [10, 18].

2 $$ {V}_{Index}=\frac{V_{UCM}/{df}_{UCM}-{V}_{ORT}/{df}_{ORT}}{V_{TOT}/{df}_{TOT}} $$

where df_UCM_ is degrees of freedom of V_UCM_ (df = 1); df_ORT_ is degrees of freedom of V_ORT_ (df = 1); V_TOT_ is the total variability (V_UCM_ + V_ORT_); df_TOT_ is degrees of freedom of V_TOT_ (df = 2); V_Index_ ranges from − 2 to 2;

3 $$ {V}_{Index}\left(Z- transformed\right)=0.5\times \mathit{\ln}\frac{2+{V}_{Index}}{2-{V}_{Index}} $$

For statistical analyses, mean data of all dependent variables (i.e., mean force, force variability, asymmetry of mean force, asymmetry of force variability, correlation coefficient, V_Index,_ V_UCM,_ and V_ORT_) were submitted to two-way mixed model (Group × Vision Condition; 2 × 2) ANOVAs with repeated measures on the last factor. Using the Shapiro-Wilk’s W test and Levene’s test [27, 28], we confirmed the normality of distribution and the homogeneity of variance assumptions for all dependent variables across group and vision conditions. For post hoc analyses, we used Bonferroni’s pairwise comparisons. All statistical analyses were performed using IBM SPSS Statistics 22 (SPSS Inc., Chicago, IL, USA) at the conventional alpha level (α = 0.05).

## Results

### Mean force, force variability, and asymmetry between hands

The two-way mixed ANOVA on mean force produced by two hands revealed a significant Group × Vision Condition (2 × 2) interaction [*F* (1,18) = 10.75; *P* = 0.004; partial η^2^ = 0.37; Table 1]. Post hoc analysis indicated that mean force in the older adult group was significantly higher in the no vision condition than the vision condition (*P* = 0.003) whereas no significant changes in mean force appeared in the young adult group (*P* = 0.25). The analysis on force variability failed to identify any significant main effects or interaction.

**Table 1:** Bilateral force control findings between young and older adults

Mean Force (N)	Old	Young	Significance
Vision	12.6 (1.2)^#^	11.5 (1.6)	Group × Vision
No Vision	13.2 (1.4)^#^	11.3 (1.5)	Interaction
Asymmetry of SD bt Hands (%)	Old	Young	Significance
Vision	121.3 (8.8)	96.7 (6.7)	Group Main Effect
No Vision	115.2 (7.2)	93.7 (6.7)	
Z-transformed RHO	Old	Young	Significance
Vision	0.18 (0.02)	0.11 (0.02)	Group Main Effect
No Vision	0.24 (0.03)	0.19 (0.02)	Vision Main Effect
Z-transformed V_Index_	Old	Young	Significance
Vision	1.38 (0.19)^#*^	2.10 (0.17)^#*^	Group × Vision
No Vision	0.75 (0.18)^#^	0.67 (0.21)^#^	Interaction

Note. Date are mean ± standard error. *Asterisk* (^*^) indicates significant difference between two groups (*P* < 0.05). *Number sign* (^#^) indicates significant difference between vision conditions (*P* < 0.05). *Abbreviations*. bt: between; SD: standard deviation; RHO: correlation coefficient; V_Index_: the index of bilateral motor synergies

Analysis on the on asymmetry of mean force data failed to show any significant main effects or interaction. However, the asymmetry analysis of force variability showed a significant Group main effect [*F* (1,18) = 5.44; *P* = 0.03; partial η^2^ = 0.23; Table 1]. Specifically, older adults revealed greater values of force variability in asymmetry than those in the young adult group when collapsed across the two vision conditions.

### Interlimb force coordination: correlation and bilateral motor synergies

The Group × Vision Condition (2 × 2) mixed ANOVA on the correlation coefficients showed two significant main effects: (a) Group: *F* (1,18) = 8.18; *P* = 0.01; partial η^2^ = 0.31 and (b) Vision Condition: *F* (1,18) = 8.80; *P* = 0.008; partial η^2^ = 0.33. Specifically, correlation coefficients in the older adult group were significantly greater than those in the young adult group when collapsed across the two vision conditions (Table 1). Both groups revealed significant higher correlation coefficients in the no vision condition (*M* ± *SE* = 0.42 ± 0.03) than those in the vision condition (*M* ± *SE* = 0.29 ± 0.03).

V_Index_ analyses revealed a significant Group × Vision Condition interaction [*F* (1,18) = 8.32; *P* = 0.01; partial η^2^ = 0.32; Table 1]. Bonferroni’s pairwise comparisons identified that V_Index_ in the older adult group was significantly less than those in the young adult group at the vision condition (*P* = 0.01), and further both groups significantly reduced V_Index_ from vision to no vision conditions (*P* < 0.01). The analysis on the V_UCM_ failed to find any significant main effects or interaction. For V_ORT_, the two-way analysis found a significant Vision Condition main effect [*F* (1,18) = 7.17; *P* = 0.02; partial η^2^ = 0.29]. Collapsed across two groups, V_ORT_ was significantly higher in the no vision condition (*M* ± *SE* = 22.96 ± 8.53%target^2^) than the vision condition (*M* ± *SE* = 4.80 ± 1.92%target^2^).

## Discussion

This study examined age-related changes in interlimb force coordination by quantifying bilateral motor synergies while manipulating visual feedback. Participants performed bilateral force control tasks at 5% of MVC with and without visual information conditions. The findings indicated that older adults exhibited greater mean force produced by two hands from the vision to no vision conditions. Across both vision conditions, the older adult group showed higher asymmetrical force variability (i.e., SD of non-dominant hand > SD of dominant hand) and revealed more positive values of correlation coefficient between forces produced by two hands than the young adult group. Finally, an index of bilateral motor synergies was significantly greater in young adults than older adults when visual information was available.

Without visual feedback, higher bilateral mean force produced by the older adults was consistent with previous findings that used unilateral force control paradigms [23, 24]. During the absence of visual feedback, forces may drift slowly away from the targeted force level because of spontaneous changes in performance [29]. Thus, the higher mean force observed after the removal of visual information may involve compensatory behaviors in dealing with potential unexpected force drift [24]. Perhaps, this compensatory action was excessive in the older adult group during bilateral force control.

In addition, as compared with the young adult group, the older adult group showed greater force variability produced by the non-dominant hands than those by dominant hands during bilateral force control across vision conditions. Despite no significant changes in the asymmetry of mean force between the two groups, our asymmetrical force variability findings indicated that older adults might experience difficulty in modulating neural noise and neuromotor drive interfering with the non-dominant hand control during bilateral force production [30, 31]. Moreover, according to the proposition of motor lateralization [32], the non-dominant hand control affected by the right hemisphere is responsible for modulating the stability of limb movements using an impedance control. These findings support the hypothesis that ageing may facilitate fewer advantages in non-dominant hands because of a decline of the right hemisphere involvement [33]. Consequently, these deficits may cause impairments in stabilizing bilateral force control.

The interlimb coordination patterns within a single-trial showed that older adults exhibited more positive correlation between the left and right hand forces than young adults, and this pattern appeared in both the vision and no vision conditions. These findings are consistent with prior reported evidence [12]. Despite the intrinsically more stable in-phase movements between hands [34], successful bilateral isometric force control was associated with more negative correlations reflecting the compensatory and less stable-anti-phase coordination [9]. Taken together, the older adult group showed less coordinated force outputs than the young adult group in a time-series while processing visual information as well as anticipating and maintaining bilateral forces around the targeted force level without visual information.

Importantly, this study found less bilateral motor synergies in the older adult group than the young adult group under the visual information condition. These findings were consistent with our hypothesis. Moreover, both groups showed a reduction in bilateral motor synergies without visual feedback. These findings indicate that deficits in interlimb coordination functions across multiple trials in the older adults [20], and the presence of visual information positively optimized motor synergies between multiple effectors (e.g., left and right hands) in the motor system. A recent study by Marchini and colleagues [21] used the UCM hypothesis for estimating coordination patterns of bilateral feet forces while executing ankle dorsiflexion movements between young and older adult groups. The results revealed that older adults exhibited fewer bilateral motor synergies in the lower extremities than young adults, and collapsed across two groups the level of bilateral motor synergies was greater in the visual feedback condition than the no visual feedback condition. Beyond the lower limb function, our findings extend impaired bilateral motor synergies across multiple trials in the upper extremities of older adults. Perhaps, an age-related neuromuscular system such as impaired visuomotor processing may cause an inability to organize and select multiple solutions in a synergic way across trials leading to affected interlimb coordination functions.

Potential neurophysiological mechanisms underlying impaired interlimb coordination in older adults include altered functions in (a) the corpus callosum and (b) the cerebellum. During bilateral movement tasks, we must address interhemispheric connections through balancing interhemispheric inhibitions between hemispheres for successful task completion [35]. Brain imaging studies reported that older adults exhibited a smaller volume of the corpus callosum and reduced white matter integrity than young adults [36]. Thus, these structural and functional changes (e.g., less interhemispheric connectivity) in the corpus callosum may lead to more functional motor impairments in interlimb coordination [37]. Another possibility is the altered cerebellar functions modulating bilateral motor synergies. The cerebellum may contribute to enhancing motor coordination and online-motor corrections as well as facilitating error-based learning across multiple trials [38]. Indeed, the cerebellar regions as well as the sensorimotor cortex in individuals who are over 60 years old served as reliable predictors of interlimb coordination performances [39, 40]. Presumably, ageing influences cerebellar functions that may directly (or indirectly) interfere with bilateral motor synergies.

Despite impaired coordination functions in the older adults as indicated by less bilateral motor synergies, we cautiously interpret these findings. Given that we did not find significant differences in force variability between the older and young adult groups, how the altered coordination patterns influenced bilateral task performance in the older adults is unclear. The low targeted force level (i.e., 5% of MVC) used for this study may limit the task performance change between the groups. Thus, future studies will investigate the relationship between bilateral motor synergies and task performance in older adults with various higher targeted force levels.

## Conclusions

In summary, the current study showed less bilateral motor synergies in the older adults than the young adults while bilaterally controlling isometric forces with visual feedback. Further, across vision and no vision conditions, the older adults produced more asymmetrical force variability and positive correlations between the two hands. Ageing may cause deficits in coordinating and selecting optimal pairs of bilateral force outputs in a synergic way across multiple trials.

## Data Availability

The datasets used and analysed during the current study are available from the corresponding author on reasonable request.

## Abbreviations

CNS: Central nervous system
MVC: Maximum voluntary contraction
ORT: Orthogonal
UCM: Uncontrolled manifold
